# Brief social presence improves delayed memory for online lecture content

**DOI:** 10.1371/journal.pone.0318149

**Published:** 2025-01-29

**Authors:** Lindsay A. Santacroce, Rachel Appiah, Margot D. Sullivan, Julia Spaniol

**Affiliations:** Department of Psychology, Toronto Metropolitan University, Toronto, Ontario, Canada; King Abdulaziz University Faculty of Medicine, SAUDI ARABIA

## Abstract

During the COVID-19 pandemic, the use of videoconferencing platforms became ubiquitous in postsecondary education around the world, making it crucial to understand how to maximize the efficacy of synchronous online classes. Given that social information can act as a motivation and improve memory, the current study tested the hypothesis that brief social presence during an online class would act as a social reward that would increase delayed memory for lecture information. Undergraduate students attended a mock synchronous class during which they viewed a pre-recorded science lecture, and social presence was manipulated by having participants turn on their cameras before and after the lecture (high social presence) or having cameras remain off during the entire class (low social presence). After 24 hours, participants completed a surprise memory test for the lecture material and a subjective experience survey about the class. As predicted, participants in the high social presence condition demonstrated higher recognition memory for the lecture content and gave higher ratings of enjoyment and feeling part of a group compared to participants in the low social presence condition. These findings suggest that enhancing social presence in virtual environments may be leveraged to boost academic performance in university students.

## Introduction

The COVID-19 pandemic caused worldwide disruptions of traditional in-class learning, causing a shift to online learning environments that has remained common in the postsecondary sector even after the pandemic [1]. Over half (54.4% as of the fall semester of 2022) of undergraduate students in the United States were taking at least one online course [2] and 47% of postsecondary administrators reported that their schools were increasing spending for online programs [3]. Online courses are well-received by students overall, with 75% of students believing that online education is equal to or better than in-person learning [3]. While postsecondary students enjoy the *convenience* of online learning [3, 4], they nonetheless express a number of pedagogical and social concerns with the online format, notably the poorer quality of instruction and the lack of community [3–6]. Immediately after the mandatory switch to all online classes in the spring of 2020, students overwhelmingly reported learning less in their classes, citing decreased motivation, ability to focus, and self-regulation as primary factors [7]. Given this, coupled with the increasing prevalence of online courses, it is crucial to understand the challenges students face with remote learning and to propose solutions for the future.

One key challenge accompanying online remote learning is the limited amount of social interaction [3–5, 7]. This challenge is critical, given that social presence, or the degree to which individuals perceive others as “real” and are able to feel connected to one another [8], has been shown to correlate positively with students’ perceived learning experience and satisfaction in remote learning environments [8, 9]. In addition to the benefits of social presence on students’ subjective perception of their courses, research has shown that higher social, cognitive, and teaching presence are predictive of higher course marks for both synchronous and asynchronous online courses [10], suggesting that social presence can also affect students’ academic performance. This could be because social interactions can affect different aspects of cognition [11–14], such that a participant’s reported degree of general social interaction positively predicts their cognitive performance [13]. Moreover, some research has suggested that perceived similarity to others in courses might increase academic performance [15]. Of note, in-lab manipulations of social interaction have shown that even brief interactions with others can affect cognition and memory. For example, just ten minutes of social interaction can increase a participant’s processing speed and working memory [13], and simple introductions with others can improve executive functioning [14]. It is possible, then, that the mere presence of others is sufficient to improve cognitive performance.

The idea that social presence affects cognition and memory is supported by the fact that processing social information, such as faces, engages reward-related brain regions such as the striatum, amygdala, ventromedial prefrontal cortex, and lateral prefrontal cortex [16]. Both anticipation and receipt of reward during learning is associated with enhanced subsequent memory, even delayed memory for unrewarded information presented close in temporal proximity to the reward [17–20]. Neuroimaging studies have demonstrated increased connectivity between the mesolimbic dopamine system and regions involved in episodic long-term memory (e.g., the hippocampus) during learning under high reward, consistent with the idea of reward-related upregulation of long-term consolidation [20–23].

Because social information is processed similarly to motivational and rewarding information [16], it is possible that simply viewing faces will elicit similar memory advantages for future information as with rewarding stimuli. That is, seeing a face might increase one’s motivational state, which could improve long-term memory consolidation during subsequent learning. This could, in turn, explain why higher social presence in online courses leads to higher grades [10]—social presence potentially acts as a reward that enhances memory for course concepts. Further, it is known that the use of cameras during synchronous online courses can improve students’ subjective sense of community [24], engagement [24, 25], and motivation [4, 26] during class. Whereas these subjective measures support the idea that social presence is related to motivation, it remains unclear if this increase in socially-driven motivation from camera use results in measurable memory improvement—and, by extension, improved test scores—similar to what is seen with reward-driven motivation [18, 19]. Moreover, a majority of previous research on camera use during synchronous online courses has been conducted by surveying students currently enrolled in online courses [24–28], introducing a potential selection bias. Finally, existing studies were not designed to differentiate among alternative causal pathways. For example, the subjective increase in motivation may be due to general social presence [29], social interactions in these courses [30], an increased feeling of accountability (or fear of judgment) when others are watching [24], or attention spreading from other classmates during the lecture [31, 32]. It is thus crucial to focus on more objective measures of course performance and to control for confounding factors when considering the motivational impact of social presence via camera usage during synchronous online courses.

Therefore, the current study specifically aimed to test whether the mere presence of faces (i.e., seeing the other participants or “classmates”) would improve memory for lecture material. Participants were randomly placed in Zoom “classrooms” in which cameras were either on or off (high vs. low social presence). To eliminate the potential for distraction by faces in the high social presence group during the lecture itself, cameras were on only before and after the lecture. It was predicted that because processing social information is similar to processing motivational information [16] and reward-driven motivation can improve delayed memory performance for unrelated items [18, 19], simply seeing the faces of other classmates prior to the lecture would increase delayed memory performance for the information presented in that lecture.

## Method

### Power and sample size justification

The current study had a single independent variable, social presence (high vs. low), which was manipulated between subjects. The key dependent variable was performance on the delayed recognition test. G*Power [33] was used to determine the target sample size. Power analysis was conducted to detect a medium effect (*d* = 0.50) for the independent samples *t*-test comparing recognition memory performance for each social presence group with 80% power and an α level of .05. These parameters resulted in a target sample size of 64 per social presence group, and thus the current study aimed to recruit a total of 128 participants.

### Participants

A total of 136 undergraduate psychology students at Toronto Metropolitan University participated for course credit. Eight participants were excluded from analysis for reasons outlined in the pre-registration: six participants failed to complete both sessions of the two-part study, and two withdrew their consent for their data to be included. An additional group of six were excluded because they attended the lecture day session with no other participants present (because of no-shows or cancellations), and thus experienced no social presence which could have biased the results. As a result of these exclusions, the final sample size included 122 participants, 61–47 women, 12 men, 2 nonbinary; *M*_age_ = 23.17, *SD*_age_ = 8.46 (two participants responded “prefer not to answer” for their age)—were assigned to the high social presence group and 61–46 women, 14 men, 1 gender-fluid; *M*_age_ = 23.66, *SD*_age_ = 9.08—were assigned to the low social presence group. All participants provided written informed consent and procedures were approved by the Research Ethics Board at Toronto Metropolitan University (#2020–427).

### Procedure

**Fig 1:**
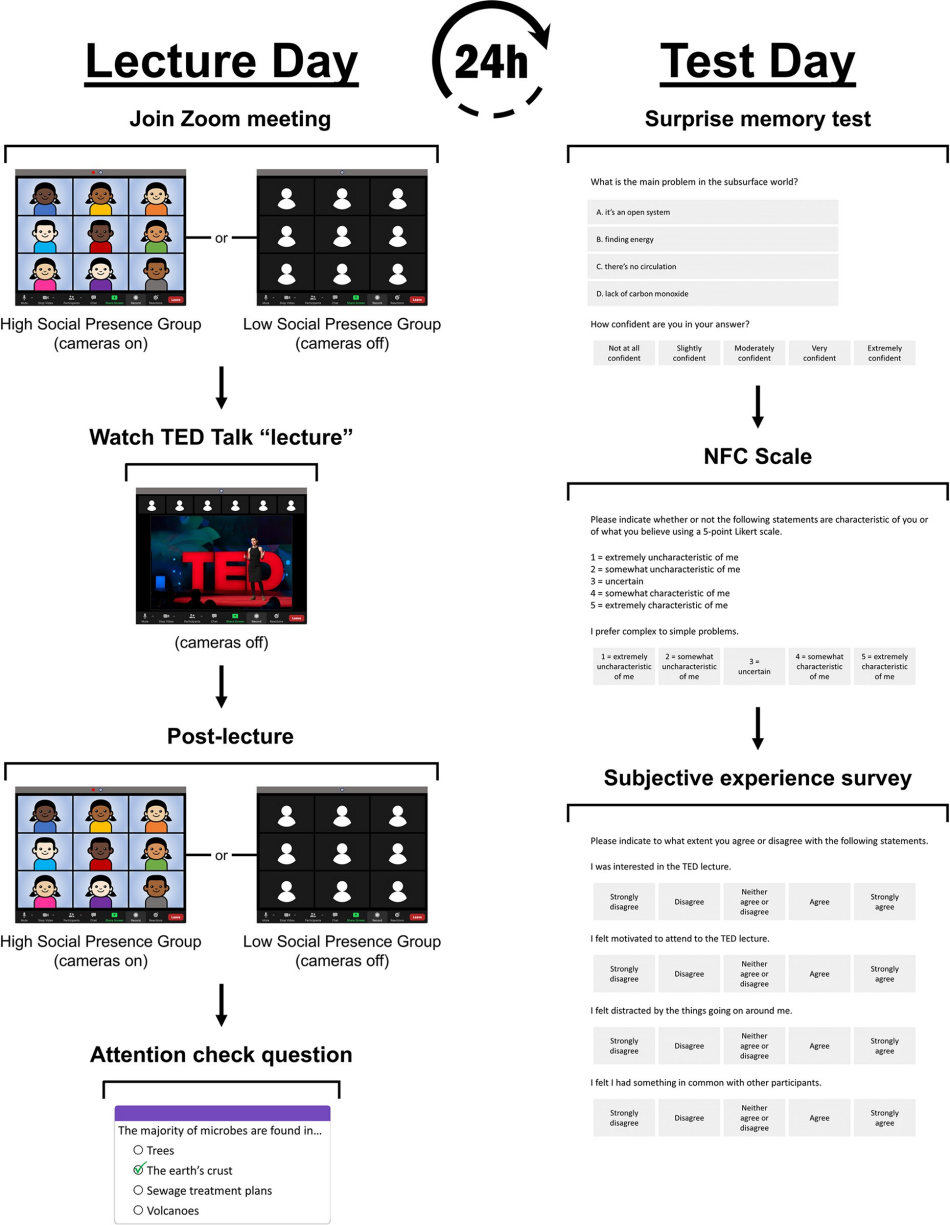
Experiment procedure. A breakdown of the experiment procedures, separated across the two days. On Lecture Day (left), participants and the researcher entered the Zoom classroom either with their cameras turned on (high social presence group) or off (low social presence group), watched the TED Talk lecture with their cameras off, then ended the class with their cameras turned on (high social presence group) or off (low social presence group). Following the Zoom class, participants completed an attention check question via a Google Form. Test Day (right) happened 24 hours after Lecture Day and included a surprise memory test on the lecture material, the Need for Cognition (NFC) scale, and the subjective experience survey with items that alluded to their opinions of the lecture, motivation, distractibility, and perceived similarity to others during the experiment.

The study hypotheses and data analysis plan were preregistered prior to data collection (https://osf.io/4bw8c; DOI: https://doi.org/10.17605/OSF.IO/4BW8C). See Fig 1 for an illustration of the study procedure. The study took place over the course of two days. The first day involved the synchronous lecture (lecture day) and the second day was a self-paced survey (test day). Participants selected from a number of time slots to partake in lecture day which, unbeknownst to them, were associated with either the high or the low social presence condition. Both conditions occurred equally often at each time of day, and up to eight participants could attend each lecture day. The actual number of participants attending each lecture ranged from two to seven (*M* = 5.52, *SD* = 1.36), and the average number of participants in the high social presence group (*M* = 5.39, *SD* = 1.32) did not differ significantly from the average number of participants in the low social presence group (*M* = 5.66, *SD* = 1.40), *t*(120) = 1.06, *p* = .289. Data collection took place during COVID-19 lockdowns when Toronto Metropolitan University courses were still exclusively online: between February 4^th^, 2021, and April 13^th^, 2021.

Participants in each social presence group attended a Zoom meeting on their scheduled lecture day, during which they first received instructions from the researcher before being shown a TED Talk lecture. All participants were muted and unable to exchange messages among each other using the chat function, although they could contact the researcher via a private message in the chat. To maintain consistency, each session was hosted by the same researcher who read the introduction and instructions from a script. The instructions primarily explained the Zoom functions that were available for participants.

Participants were then shown a short video “lecture” on an advanced biology topic. The lecture video was a TED Conference presentation by Dr. Karen Lloyd titled “The mysterious microbes living deep inside the earth—and how they could help humanity” [34]. In the video, Dr. Lloyd discusses, in detail, microbes in the earth’s subsurface, a complex topic from the field of microbiology and biodiversity. The video was selected to ensure that most participants (students from a psychology course) would have little or no prior knowledge on the topic. The video duration was 13 minutes and 52 seconds and was presented through the researcher’s shared screen during the Zoom meeting. Following the lecture, participants answered an attention check question, "The majority of microbes are found in…", by selecting one of four multiple-choice answers provided via a Google Form. The lecture day sessions lasted about 30 minutes in total.

The key manipulation for the social presence groups was their camera usage at the beginning and end of the lecture day Zoom meeting. Participants in the high social presence condition entered and exited the Zoom session with their cameras on, whereas participants in the low social presence condition completed the entire session with their cameras off. Moreover, the researcher also began and ended the meeting with their camera turned on in the high social presence condition, but left their camera off in the low social presence sessions. Thus, participants in the high social presence group were able to see the other Zoom participants in their session prior to and following the lecture. Notably, because the experiment took place on Zoom, participants in both groups saw the same video tiles that were either filled with participants’ videos in the high social presence group or the default Zoom avatars in the low social presence group, which controlled the visual display during the experiment. Critically, following the instructions, participants in the high social presence group were prompted to turn off their cameras during the lecture, and so both social presence groups had their cameras off while viewing the video lecture. This ensured that the encoding episode was identical for both levels of the social presence manipulation. That is, any potential group effects would be the result of briefly seeing the faces of other participants *before and after* learning the materials, and not because other participants affected attentiveness *during* the lecture [32]. After the video lecture, the researcher prompted participants in the high social presence condition to turn their cameras back on prior to an attention check question on the lecture video.

Sessions on testing day took place 24 hours after the completion of sessions on lecture day and included a survey that had to be completed within 24 hours. Participants received a link to a Qualtrics survey that included a demographic questionnaire, the Need for Cognition (NFC) Scale [35], a subjective experience survey, and a multiple-choice memory test on the lecture from the previous day. The multiple-choice test was used to assess participants’ delayed memory for the video content. The questions (e.g., "What is the main problem in the subsurface world?") were specific to the video lecture, and thus, it was unlikely that participants were able to use any prior knowledge to answer the test questions. The test contained 16 multiple-choice questions with four response options. Participants also reported their confidence in each of the answers using a 5-point Likert scale, with ‘5’ signifying the highest level of confidence.

In the subjective experience survey, participants were asked to rate statements about their perceived experience during the lecture and overall experiment. The survey items alluded to their opinions of the lecture (e.g., “I was interested in the TED lecture”), their motivation (e.g., “I felt motivated to attend to the TED lecture”), their distractibility (e.g., “I felt distracted by the things going on around me”), and their perceived similarity to others during the experiment (e.g., “I felt I had something in common with other participants”). Ratings were made on a 5-point Likert scale, with ‘5’ indicating that the statement applied to them very well and ‘1’ indicating that the statement does not apply to them at all. Finally, the NFC Scale was administered to assess each participant’s tendency to engage in and enjoy thinking (e.g., “I prefer complex to simple problems”), also rated on a 5-point Likert scale. The NFC was administered to make sure any differences were because of the manipulated camera use, and not because of individual participants’ typical interest in learning.

## Results

All analyses were conducted in JASP [36]. To test whether social presence affected participants’ perceived experience during the lecture, all ratings from the subjective experience survey were subjected to two-tailed independent sample *t*-tests comparing the high social presence group to the low social presence group. We were primarily interested in the survey items categorized as being related to participants’ opinions of the lecture, motivation, distractibility, and perceived similarity to others. See Table 1 for a list of all 17 survey items in the subjective experience survey, their categorizations, and each corresponding *t*-test output. With regards to their overall opinions of the lecture, the results yielded no significant differences between the two groups in terms of their interest in the lecture (*p* = .467) or how engaging they found the lecture (*p* = .074). However, participants in the high social presence group reported that they enjoyed the lecture session more than those in the low social presence group (*p* = .025). Participants in the high social presence group were not more motivated to attend the lecture (*p* = .196) and did not feel that they had more in common with the other participants (*p* = .826), the researcher (*p* = .911), or the lecturer (*p* = 1.000). In addition, there were no differences in participants’ distractibility during the lecture (*p*s > .057). Participants in the high social presence group did, however, report feeling more like they were a part of a group (*p* = .017) and present with the other participants (*p* = .001). The results from the subjective experience survey are thus generally similar for both groups, but participants in the high social presence group overall enjoyed the lecture day session more and felt like a part of a group.

**Table 1:** Output from the subjective experience survey *t*-tests by survey item.

Survey items	LSP		HSP		*t*(120)	*p*	*d*
	*M*	*SD*	*M*	*SD*			
**Items about opinions of the lecture**							
I was interested in the TED lecture.	3.36	1.16	3.51	1.07	-0.73	.467	-0.13
I felt like the lecture was engaging.	3.13	1.25	3.49	0.94	-1.81	.074	-0.33
I enjoyed Session 1.	3.39	0.88	3.74	0.79	-2.27	.025*	-0.41
**Items about motivation**							
I felt motivated to attend to the TED lecture.	2.92	1.05	3.16	1.04	-1.30	.196	-0.24
**Items about distractibility**							
I was able to concentrate on the TED lecture.	2.95	1.22	3.36	1.14	-1.92	.057	-0.35
I was able to block out most distractions.	2.84	1.24	3.08	1.23	-1.10	.274	-0.20
I felt distracted by my thoughts.	3.41	1.23	3.38	1.17	0.15	.880	0.03
I felt distracted by the things going on around me.	3.28	1.29	2.92	1.14	1.63	.105	0.30
**Items about similarity to others**							
I felt I had something in common with other participants.	3.05	0.85	3.08	0.80	-0.22	.826	-0.04
I felt I had something in common with the researcher.	3.02	0.79	3.00	0.84	0.11	.911	0.02
I felt I had something in common with the lecturer in the video.	3.02	1.06	3.02	0.99	0.00	1.000	0.00
**Quality control items**							
I felt like I was part of a group yesterday.	2.54	1.07	3.02	1.09	-2.43	.017*	-0.44
I felt like I was present with the other Zoom participants.	2.48	1.03	3.12	1.13	-3.28	.001*	-0.59
I felt curious to see the other participants.	3.16	1.08	3.02	1.15	0.73	.467	0.13
I enjoyed being present with other participants during Session 1.	3.16	0.88	3.39	0.94	-1.40	.165	-0.25
In general, I prefer to see the other attendees that I am in a Zoom call with.	3.26	1.08	2.97	1.21	1.42	.158	0.26
I am interested in environmental science topics.	3.02	1.22	3.03	1.25	-0.07	.942	-0.01

LSP, low social presence group; HSP, high social presence group.

*p < .05.

To test for an effect of social presence on immediate memory performance, a chi-squared test of independence was conducted on the percentage of participants who accurately responded to the post-lecture question on lecture day. The results revealed a significant difference between the high social presence and low social presence groups, *X*^2^(1) = 4.17, *p* = .041, ɸ = 0.19, where participants in the high social presence group were correct more often (91.80% vs. 78.69%). This suggests that social presence increases attention toward and immediate memory for the lecture topic, despite them not self-reporting feeling less distracted than those in the low social presence group.

**Fig 2:**
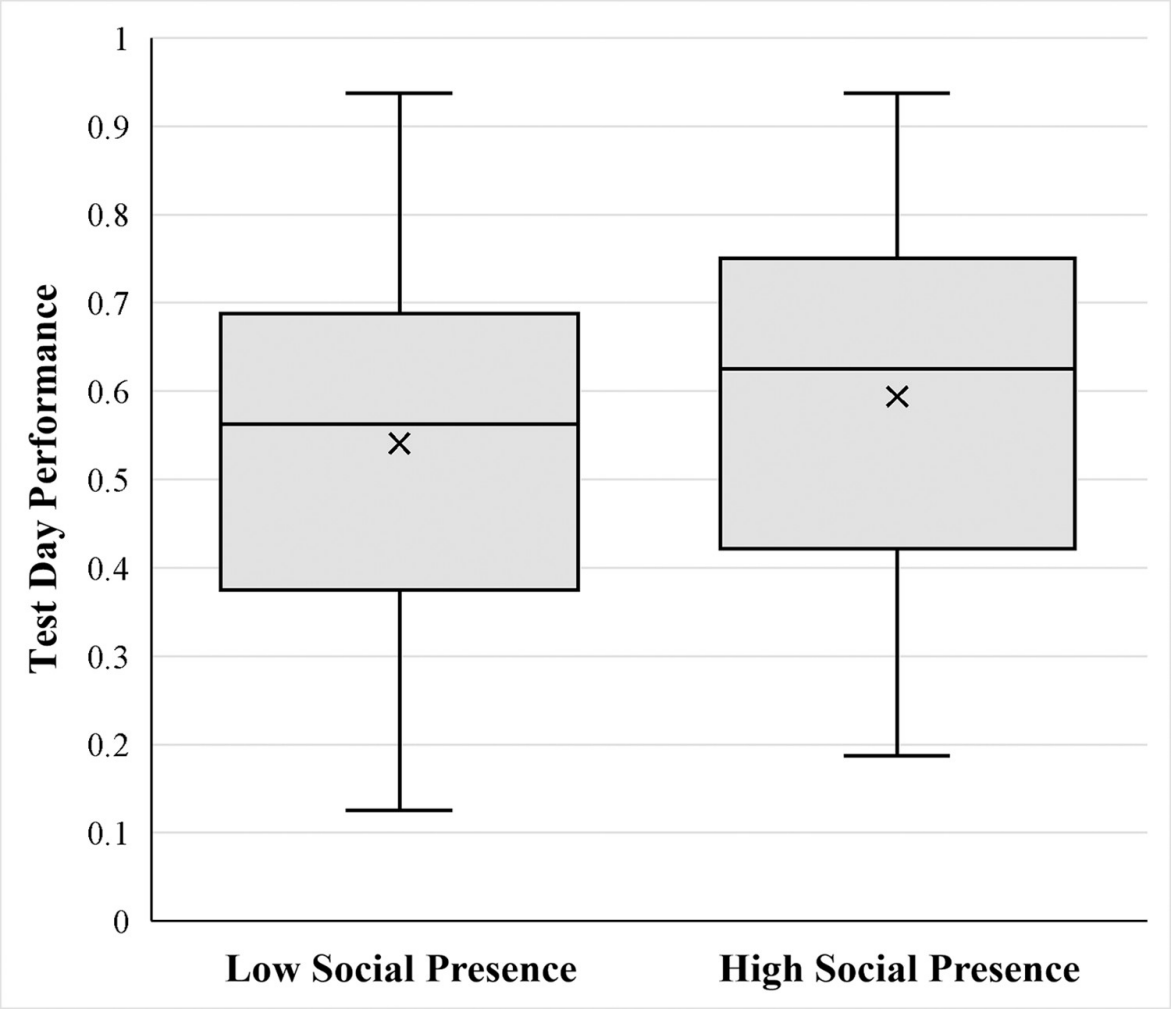
Test day quiz performance by social presence group. Average performance on the test day multiple choice memory test, separated by social presence groups.

To test for an effect of social presence on delayed recognition memory performance, a two-tailed independent sample *t*-test on the testing day quiz accuracy was conducted (Fig 2). The results revealed a significant difference between the groups for the testing day quiz, with higher performance in the high social presence group (*M* = 0.61, *SD* = 0.19) compared to the low social presence group (*M* = 0.53, *SD* = 0.21), *t*(120) = 2.31, *p* = .022, *d* = 0.42. This effect remained significant even when including NFC scores as a covariate in an analysis of variance (ANOVA), *F*(1, 119) = 6.43, *p* = .012, η_p_^2^ = 0.05, suggesting that individual differences in general interest in learning did not explain the effect of social presence on test accuracy, although the NFC score did yield a significant main effect on overall performance, *F*(1, 119) = 22.27, *p* < .001, η_p_^2^ = 0.16. Exploratory correlation analysis, as described in the preregistration, examined whether participants’ perceived similarity correlated with their overall performance, and found that their perceived similarity with the lecturer (Dr. Karen Lloyd) positively correlated with test performance, *r*(120) = 0.20, *p* = .027, but the same correlation was not evident with their perceived similarity with the other participants, *r*(120) = -0.01, *p* = .892, nor the researcher, *r*(120) = 0.12, *p* = .176. However, controlling for perceived similarity with the lecturer as a covariate in an ANOVA did not alter the significant main effect of social presence on performance, *F*(1, 120) = 5.35, *p* = .022, η_p_^2^ = 0.04, confirming that this correlation between perceived similarity with the lecturer and test day performance cannot explain the effect of social presence on test day performance.

The current study also ran additional post-hoc exploratory analyses that were not preregistered. First, Bayesian statistics were conducted alongside the main hypothesis tests, which provided marginal support for the effect of social presence on the day 1 attention check performance (BF_10_ = 2.36), the memory test performance (BF_10_ = 2.11), and the significant items from the subjective experience survey (BF_10_s = 1.92–21.75). Moreover, because research has suggested gender differences in social and learning preferences [37–39], exploratory analysis was conducted to test for gender differences in the current results. A 2 (social presence group: high vs. low) × 2 (gender: men vs. women; three nonbinary/gender-fluid participants were excluded from these analyses) ANOVA was conducted on each item from the subjective experience survey, as well as on the delayed memory test performance. The only outcome measure that yielded a significant gender × social presence group interaction with gender was the “I felt distracted by the things going on around me” item, *F*(1, 115) = 9.46, *p* = .003, η_p_^2^ = 0.08, such that women were more distracted by their environment than men in the low social presence group, *t*(118) = 3.03, *p* = .016, *d* = 0.92, and women were more distracted by their environment in the low social presence group compared to the high social presence group, *t*(118) = 2.91, *p* = .022, *d* = 0.60. Note that these gender difference results are underpowered due to the uneven samples (47 women and 12 men in the high social presence group, 46 women and 14 men in the low social presence group) and more research would need to be conducted to draw strong conclusions. Finally, a correlation was ran to determine if the degree to which a participant enjoyed the lecture predicted their performance on the memory test, which yielded a significant positive correlation, *r*(120) = 0.25, *p* = .006.

## Discussion

The current study tested the hypothesis that social presence in an online classroom can improve delayed memory for subsequent lecture content using a mock online lecture where participants either entered a Zoom “classroom” with their cameras turned on (high social presence group) or off (low social presence group). Following the lecture, both groups completed an attention check question immediately following a TED Talk lecture, and 24 hours later completed a subjective experience survey and a surprise test on the lecture materials.

The results from a subjective experience survey, which provided information about their opinions of the lecture, motivation, distractibility, and perceived similarity to others during the experiment, revealed that participants in the high social presence group enjoyed their experimental “class” session more than participants in the low social presence group and felt more like they were part of a group and present with others. These findings support the effectiveness of our social presence manipulation, and they suggest that webcam use during video lectures may bolster feelings of cohesion. This result aligns with previous research highlighting the importance of social presence during online courses on students’ perceived learning experience and satisfaction [8, 9], and showing that camera use during online Zoom classes increases students’ sense of community [24]. Surprisingly, participants in the high social presence group did not feel more motivated to attend the lecture, which contradicts research suggesting that camera use during synchronous online learning increases motivation [4, 26]. This also challenges our hypothesis that high social presence has a similar motivational impact as rewards. However, because the session took place in an experimental setting with a one-time lecture on a topic not chosen by the participants, there may have been lower investment in learning the content, which may have impacted their motivation to attend the lecture). Nevertheless, the current study was more interested in the resulting memory advantages from the potential motivational impact of faces, which might only reveal itself in the delayed memory test, and not in participants’ subjective opinions.

Participants in the high social presence group were also more likely to accurately respond to the immediate attention check question, suggesting that they may have focused on the lecture more than those in the low social presence group. This, however, was not reflected in their subjective experience survey responses, where there were no group differences in how they *perceived* their distractibility or motivation during the lecture. Their perceived distractibility or motivation was also inconsistent with previous research suggesting that students feel more engaged [24, 25] and motivated [4, 26] during online courses when their cameras were turned on. However, this inconsistency is less surprising when considering that the current study had a one-time mock lecture, rather than these previous studies who surveyed students actively enrolled in an online class that they regularly attended [4, 24–26], which may have affected how participants prioritized their attentiveness.

Most importantly, the current results revealed that participants in the high social presence group performed better on the delayed lecture quiz compared to the low social presence group, suggesting that they had better delayed memory for the topics discussed in the lecture from the previous day. This key finding was consistent with the hypothesis that seeing faces would act as a social reward [16, 40–42], and that this reward presented close in temporal proximity to an encoding episode would promote long-term memory consolidation, with hippocampus-dependent memory consolidation as a possible mechanism [18, 19, 21]. This, in turn, would yield increased delayed memory performance on the lecture topics, similar to the memory benefits of processing rewards [18, 19]. This finding supports the idea that social information (i.e., faces) is processed similar to rewarding or motivational information [16], and thus have a similar effect on memory for subsequent information [18, 19].

In addition, because the current study did not conflate the manipulated social presence with the memorizing event by having participants in both groups turn of their cameras during the TED Talk lecture, it minimizes the possibility of other explanations for the results such as distractions, increased motivation, fear of being judged, or any other outside factor brought about by camera use while attending to the lecture [27]. Moreover, this supports previous research suggesting that even brief interactions with others can affect cognition and memory [13, 14] because participants only experienced minimal social presence for a short amount of time by merely seeing others’ faces before and after the lecture. Future research could further manipulate this brief social interaction to have participants either see faces only before the lecture *or* only after the lecture to test if there is a specific time relative to the memorizing event that this social presence improves memory.

To ensure that the current results were not driven by the individual participants’ general intrinsic motivation to learn, they also completed the NFC scale on test day. While the NFC scores did play a role in overall memory performance, the current results revealed that they did not significantly modulate the effect of social presence on delayed recognition memory. Moreover, exploratory analyses compared participants’ test scores to their perceived similarity to the lecturer, researcher, and other participants. The results revealed a significant positive correlation between participants’ scores and their perceived similarity to the lecturer (Dr. Karen Lloyd), but not to the researcher or other participants. However, this measure also did not modulate the effect of social presence on delayed recognition memory. These findings rule out some aspects of individual differences that might explain the results, although future exploration of other trait variables might be beneficial.

The current study had several limitations. First, the TED Talk video on lecture day was only about 14 minutes long. While this lecture length is similar to those used in other studies of online learning [e.g., 43–49], it may not be representative of typical classroom lectures which often last significantly longer. Moreover, the delayed memory test took place only one day after the lecture, which also might be less representative of typical classes that often test students’ knowledge weeks after they initially learn the topic. Future research should consider a more longitudinal approach to see if these memory benefits would extend past 24–48 hours. Finally, the current sample consisted of only psychology majors, a majority of whom were women, thus limiting the generalizability of the current findings.

Overall, the current study highlights the benefits of brief social presence on delayed memory performance, using a simple manipulation of having video cameras on or off prior to a video “lecture”. The results support the hypothesis that brief instances of social information (i.e., faces) can have similar beneficial effects on memory as motivational information [18, 19], providing additional evidence that the two types of information are processed similarly [16]. In addition, given that online education will likely continue to grow [1], the current results could benefit universities and instructors by encouraging the use of video cameras to improve the effectiveness of online courses.
